# A lysosomal escape‐enabled endoplasmic reticulum‐targeting BODIPY photothermal agent for enhanced tumor ablation

**DOI:** 10.1002/smo2.70073

**Published:** 2026-06-24

**Authors:** Shaoyang Shi, Yanbing Cao, Jiexuan Zuo, Qiushi Li, Xiaolong Zeng, Jianjun Du, Jiangli Fan, Wen Sun, Xiaojun Peng

**Affiliations:** ^1^ State Key Laboratory of Fine Chemicals Frontiers Science Center for Smart Materials Oriented Chemical Engineering Dalian University of Technology Dalian China; ^2^ Ningbo Institute of Dalian University of Technology Ningbo China

**Keywords:** BODIPY, endoplasmic reticulum targeting, lysosomal escape, nanomedicine, photothermal therapy

## Abstract

Photothermal therapy (PTT) has emerged as a promising minimally invasive strategy for tumor treatment due to its high selectivity, minimal drug resistance, and precise controllability. However, the efficacy of PTT is often limited by the inability of photothermal agents (PTAs) to effectively target subcellular organelles after cellular uptake. In this study, we designed and synthesized an endoplasmic reticulum (ER)‐targeting BODIPY‐based photothermal agent (ER‐BDP) by functionalizing a trifluoromethyl‐substituted BODIPY scaffold with a p‐toluenesulfonyl group. The resulting ER‐BDP nanoparticles (ER‐BDP NPs), co‐assembled with DSPE‐PEG_2000_‐Biotin, exhibit excellent near‐infrared absorption (λmax = 780 nm), high photothermal conversion efficiency (80.3%), and efficient tumor targeting via the enhanced permeability and retention (EPR) effect. Importantly, ER‐BDP NPs demonstrate the ability to escape from lysosomes and specifically accumulate in the ER, where localized photothermal heating induces severe ER stress and apoptosis. In vitro and in vivo studies confirm that ER‐BDP NPs effectively ablate tumor cells under mild laser irradiation (760 nm, 500 mW cm^−2^) while showing negligible systemic toxicity. This work provides a rational design strategy for organelle‐targeting photothermal agents and highlights their potential for enhanced tumor therapy.

## Graphical abstract

To advance precision cancer therapy, we engineered ER‐BDP NPs, a self‐assembled nanomedicine designed for selective ER localization. By integrating organelle targeting with localized laser activation, this agent mediates precision‐guided tumor destruction in vitro and in vivo while reducing off‐target effects, effectively balancing therapeutic efficacy and biosafety.

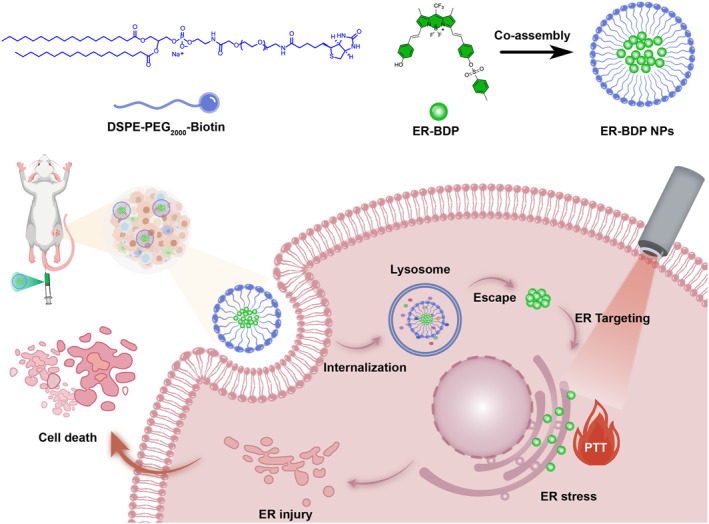

## 1 INTRODUCTION

Photothermal therapy (PTT), as a tumor treatment strategy with excellent spatiotemporal controllability, has attracted extensive attention in recent years.[^1^, ^2^, ^3^] Its fundamental principle relies on photothermal conversion agents (PTAs) that absorb near‐infrared (NIR) light and convert it into heat, thereby enabling localized ablation of tumor tissues.[^2^, ^3^, ^4^, ^5^] However, conventional PTT relies on the nonspecific diffusion of heat, typically requiring relatively high temperatures (>50°C) to achieve effective tumor cell eradication.[^1^, ^2^, ^6^] This not only causes unavoidable collateral damage to surrounding normal tissues but also compromises therapeutic precision.[^6^, ^7^]

Recent studies suggest that the efficacy of PTT does not solely originate from direct thermal destruction,[^8^, ^9^] but is closely associated with the activation of intracellular stress responses.[^7^, ^10^, ^11^] In this context, heat primarily functions as a trigger for stress signaling networks that induce programmed cell death.^[12]^ However, such responses are generally induced passively under bulk temperature elevation and lack precise spatial regulation at the subcellular level,[^13^, ^14^] thereby necessitating excessive thermal input to achieve sufficient cytotoxicity.[^15^, ^16^, ^17^] Therefore, decoupling therapeutic efficacy from hyperthermia and achieving efficient tumor cell killing through precise modulation of intracellular stress processes has become a central challenge in PTT.[^9^, ^18^, ^19^, ^20^]

Among intracellular organelles, the endoplasmic reticulum is highly sensitive to external stimuli because of its roles in protein folding and calcium homeostasis.[^21^, ^22^] Subtle perturbations can induce ER stress and activate the unfolded protein response and downstream cell death pathways.[^10^, ^11^, ^23^, ^24^] Compared with strategies that rely on nonspecific heating, precise delivery of photothermal effects to the ER can amplify stress responses under low thermal input and markedly enhance tumoricidal efficacy.[^23^, ^25^, ^26^]

Based on the above considerations, the development of photothermal molecular systems that combine high photothermal conversion efficiency (PCE) with precise subcellular localization has become a key strategy to overcome the limitations of conventional PTT.[^27^, ^28^] However, most existing photothermal agents lack effective organelle‐targeting capability within cells and are prone to sequestration in non‐critical compartments such as lysosomes, thereby limiting their ability to act on essential organelles like the ER and restricting their therapeutic potential under mild photothermal conditions.[^29^, ^30^] BODIPY‐based dyes, owing to their excellent photophysical properties and highly tunable molecular structures, have demonstrated considerable promise in photothermal therapy.[^6^, ^7^, ^11^] Nevertheless, conventional BODIPY systems still exhibit limited subcellular specificity, making it challenging to achieve efficient targeting of defined organelles.^[31]^ Therefore, endowing BODIPY scaffolds with organelle‐specific targeting capability through rational molecular engineering, while preserving their superior photothermal performance, is of significant importance for enhancing the precision and therapeutic efficacy of PTT.[^32^, ^33^, ^34^]

**SCHEME 1:**
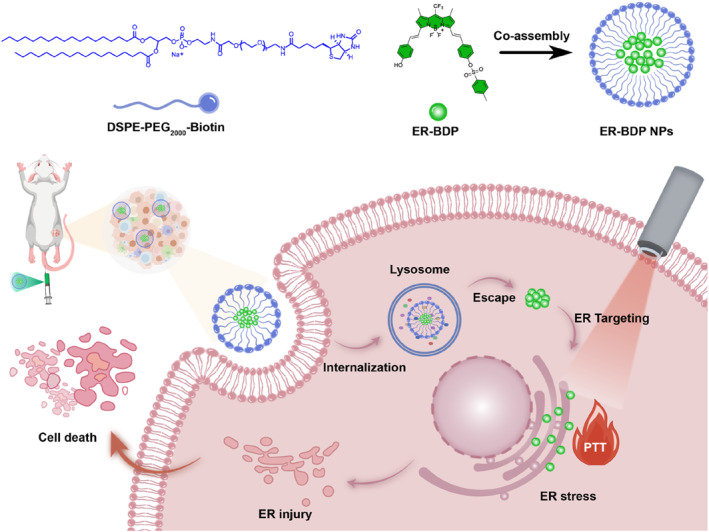
Schematic of endoplasmic reticulum‐targeting BODIPY‐based photothermal agent nanoparticles synthesis and their lysosomal escape‐mediated intracellular delivery to the endoplasmic reticulum enabling precise photothermal therapy for enhanced tumor ablation.

In this context, we rationally designed and constructed an ER‐targeted BODIPY‐based photothermal molecular system. By precisely delivering the photothermal effect to the ER, this platform markedly amplifies ER stress and its downstream cell death signaling pathways, thereby enabling efficient tumor ablation under mild photothermal conditions (Scheme 1). This strategy is expected to overcome the intrinsic reliance of conventional PTT on high temperatures, facilitating a paradigm shift from “bulk thermal damage” to “subcellularly precise regulation” and offering new insights for the mechanistic optimization and clinical translation of photothermal therapy.

### 1.1 Photophysical properties and morphological characterization of ER‐BDP NPs

**FIGURE 1:**
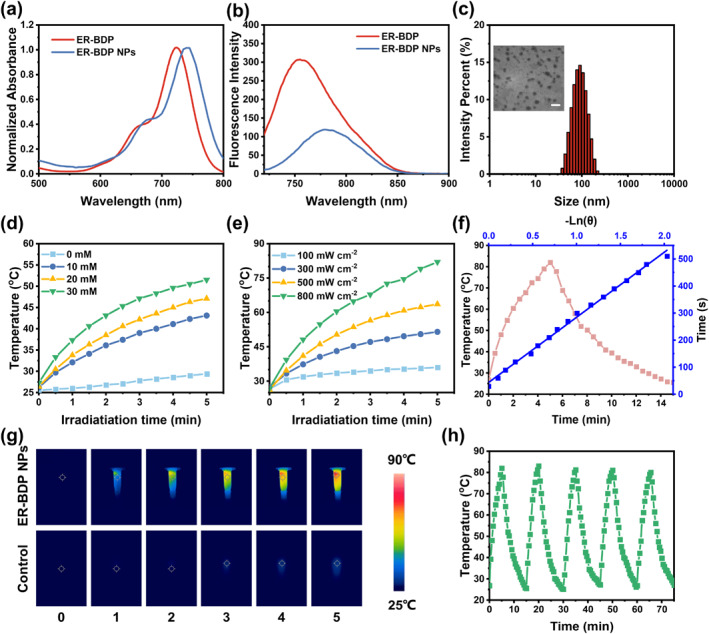
(a) UV‐Vis absorption spectra and (b) fluorescence emission spectra of ER‐BDP and ER‐BDP NPs. (c) Hydrodynamic diameter and transmission electron microscopy of ER‐BDP NPs. Scale bar: 200 nm. (d) Photothermal conversion performance of ER‐BDP NPs at different concentrations (0–30 μM) under 760 nm laser irradiation (300 mW cm^−2^). (e) Photothermal conversion performance of 30 μM ER‐BDP NPs under 760 nm laser irradiation with different laser power densities (100–800 mW cm^−2^). (f) Time constant for heat transfer of the ER‐BDP NPs system, calculated from the linear time data during system cooling and the negative natural logarithm of the system's driving force temperature. (g) Photothermal imaging of ER‐BDP NPs under 760 nm laser irradiation (300 mW cm^−2^, 5 min). (h) Photothermal stability of ER‐BDP NPs during five consecutive heating‐cooling cycles (30 μM, 800 mW cm^−2^). ER‐BDP NPs, endoplasmic reticulum‐targeting BODIPY‐based photothermal agent nanoparticles.

The UV‐Vis absorption spectra of endoplasmic reticulum‐targeting BODIPY‐based photothermal agent (ER‐BDP) and ER‐BDP nanoparticles (ER‐BDP NPs) were first tested. The absorption and emission wavelengths of ER‐BDP and ER‐BDP NPs were measured. The maximum absorption wavelength of ER‐BDP was found to be 740 nm, while that of ER‐BDP NPs encapsulated with DSPE‐PEG_2000_‐Biotin was 780 nm, ER‐BDP NPs exhibit a red‐shift of approximately 40 nm compared with ER‐BDP. This phenomenon can be attributed to the partial J‐aggregation of ER‐BDP within the nanoparticles, which results in a certain extension of the absorption wavelength and is thus favorable for the application of ER‐BDP NPs in subsequent deep‐tumor PTT (Figure 1a). ER‐BDP NPs displayed significantly quenched fluorescence relative to free ER‐BDP (Figure 1b). This phenomenon not only reflects the fluorescence quenching effect induced by the ICT arising from the D‐π‐A structural design but also confirms that ER‐BDP indeed aggregates in the nanoparticles, thus leading to the attenuation of its fluorescence. The weakened radiative transition (i.e., fluorescence) of ER‐BDP NPs indirectly enhances their non‐radiative transition, which facilitates the dissipation of energy in the form of heat and thus improves their photothermal conversion capability as a PTA.

Transmission electron microscopy (TEM) images revealed that the as‐prepared ER‐BDP NPs exhibited a uniform spherical morphology with an average diameter of 85 nm, while dynamic light scattering (DLS) measurements showed a hydrodynamic diameter of 100 nm (Figure 1c). Furthermore, the nanoparticles maintained a monodispersed state in aqueous solution for more than 7 days without any obvious changes in the average particle size or significant aggregation observed (Supporting Information S1: Figure S2). The particle size measured by DLS was larger than that observed in TEM images, which arises from the intrinsic difference between the hydrodynamic diameter and the physical diameter of dried nanoparticles. When dispersed in an aqueous environment, the hydrophilic PEG shell of the nanoparticles interacts favorably with water molecules, thus resulting in a slightly larger measured size compared with the actual physical diameter. Nevertheless, the particle size of the nanoparticles fell within the range of 50–200 nm regardless of the environment, which ensures their efficient accumulation at tumor sites via the EPR effect and the targeting capability of biotin.

### 1.2 Photothermal performance of ER‐BDP NPs

The photothermal conversion capability of ER‐BDP NPs was investigated subsequently. Under the same experimental conditions, the aqueous dispersion of ER‐BDP NPs (30 μM) was irradiated with a 760 nm laser at a power density of 300 mW cm^−2^ for 5 min, leading to a temperature increase of 24.2°C. Furthermore, the temperature increases diminished progressively as the concentration of ER‐BDP NPs decreased. In contrast, the pure water control group only exhibited a negligible temperature elevation (Δ*T* = 3.9°C) (Figure 1d). When the aqueous dispersion of ER‐BDP NPs (30 μM) was irradiated with a 760 nm laser at varying power densities for 5 min, the experimental results indicated that the temperature of the dispersion increased significantly with the elevation of laser power density (Figure 1e). The PCE of ER‐BDP was calculated to be 80.3% (Figure 1f). Specifically, upon irradiation at a power density of 800 mW cm^−2^, the temperature of the dispersion exceeded 80°C within 5 min, whereas no obvious drastic temperature increase was observed in the control group (Figure 1g). Collectively, these results demonstrate that the heat generation can be effectively regulated by adjusting the concentration of ER‐BDP NPs, laser power density, and irradiation duration, indicating that the temperature rise primarily depends on these key parameters. After five heating‐cooling cycles, the ER‐BDP NPs still exhibited excellent thermal stability and photostability (Figure 1h). This superior stability is attributed to the remarkable chemical stability of ER‐BDP endowed by its D‐π‐A structural design, demonstrating that the nanoparticles maintain favorable stability and sustainable PCE even under continuous laser irradiation.

### 1.3 In vitro cellular uptake and organelle localization assay

Prior to photocytotoxicity evaluation, the cellular uptake of ER‐BDP NPs in 4T1 cells was assessed by confocal laser scanning microscopy (CLSM) to determine the optimal incubation time based on intracellular fluorescence intensity. The intracellular fluorescence intensity of ER‐BDP NPs in 4T1 cells increased over time and reached a maximum at 2 h. Subsequently, the intracellular fluorescence signal remained at this high intensity for an additional 2 h (Supporting Information S1: Figure S3). This indicates that cellular drug uptake reached its maximum at 2 h and that the drug could be retained within the cells for an extended period. Thus, providing a favorable time window for laser irradiation post ER‐BDP NP administration.

**FIGURE 2:**
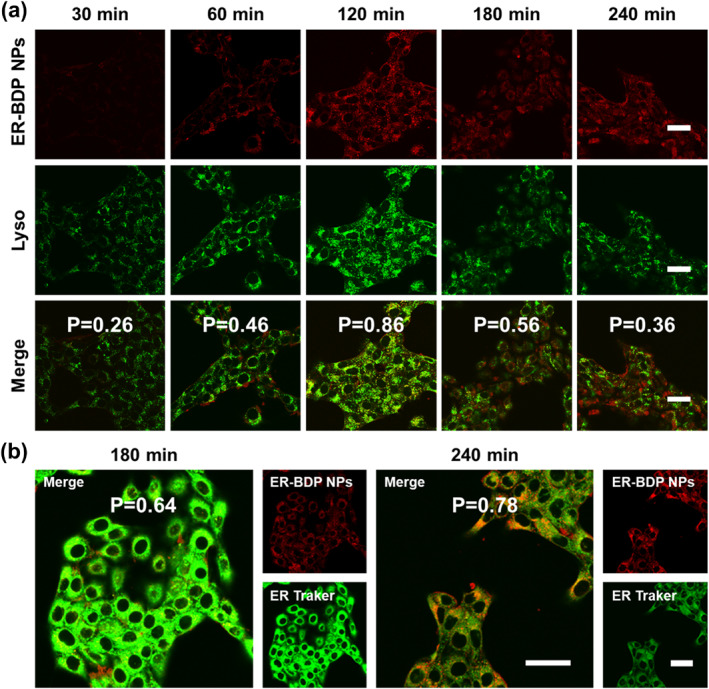
Fluorescence images of endoplasmic reticulum‐targeting BODIPY‐based photothermal agent nanoparticles colocalized with lysosomes (a) and endoplasmic reticulum (b). Scale bar: 50 μm.

To verify whether ER‐BDP NPs could escape from lysosomes and ultimately target the ER to induce ER stress, colocalization experiments of ER‐BDP NPs with ER and lysosomes were performed using CLSM. After separate incubation with Lysotracker Green and ER‐BDP NPs, 4T1 cells were imaged by CLSM at set intervals to calculate colocalization coefficients, allowing assessment of colocalization and lysosomal escape. The colocalization coefficient between ER‐BDP NPs and lysosomes increased to a peak of 0.86 at 2 h and then declined to 0.36 by 4 h (Figure 2a), indicating initial lysosomal accumulation followed by escape. In parallel, colocalization with the ER was also examined, with the coefficient rising to 0.78 by 4 h (Figure 2b). These findings demonstrate that ER‐BDP NPs undergo lysosomal escape and subsequently migrate to the ER, achieving specific ER targeting.

Together, these data confirm that ER‐BDP NPs first accumulate in lysosomes, then escape and relocate to the ER, achieving targeted delivery to this organelle.

### 1.4 In vitro cytotoxicity assay

**FIGURE 3:**
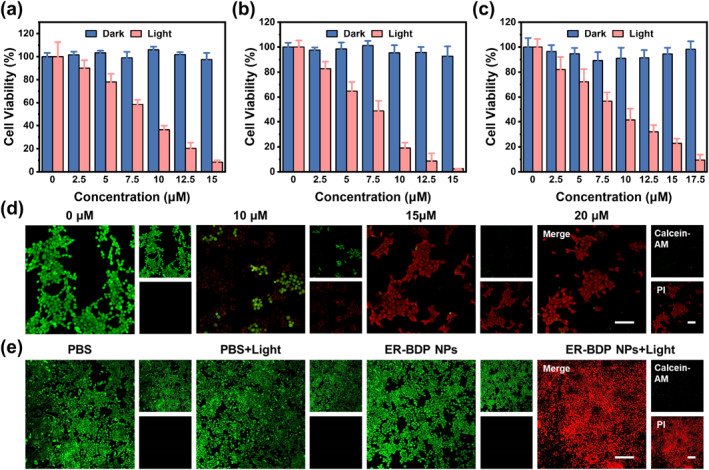
Cell viability rates of 4T1 (a), A549 (b), and MCF‐7 (c) cells treated with ER‐BDP NPs at different concentrations under light irradiation or dark conditions. (d) Fluorescence images of 4T1 cells stained with live/dead assay in the presence of ER‐BDP NPs at various concentrations. Scale bar: 100 μm (e) Fluorescence images of 4T1 cells stained with live/dead assay under different treatment conditions. For all light‐irradiated groups, cells were exposed to a 760 nm laser at 500 mW cm^−2^ for 10 min. Scale bar: 200 μm. ER‐BDP NPs, endoplasmic reticulum‐targeting BODIPY‐based photothermal agent nanoparticles.

To first evaluate the dark toxicity and phototoxicity of ER‐BDP NPs across different concentrations, the MTT assay was performed on three tumor cell lines: 4T1, A549, and MCF‐7. Under light irradiation (760 nm, 500 mW cm^−2^, 10 min), ER‐BDP NPs exhibited a distinct concentration‐dependent phototoxic effect. Specifically, at 15 μM, cell viability in 4T1 and A549 cells dropped below 10%, whereas MCF‐7 cells required a slightly higher concentration of approximately 17.5 μM to achieve comparable cytotoxicity (Figure 3a–c). The half‐maximal inhibitory concentration (IC50) of ER‐BDP NPs under laser irradiation was calculated to be 8.76 μM for 4T1 cells, 7.29 μM for A549 cells, and 9.44 μM for MCF‐7 cells (Supporting Information S1: Figure S4). Under dark conditions, the cell viability remained above 90% for all three cell lines, confirming the excellent biocompatibility of ER‐BDP NPs. Based on these results, the 4T1 cell line and a concentration of 15 μM were selected for subsequent experiments.

The concentration‐dependent cytotoxicity was further visualized using the Calcein‐AM/PI live/dead staining assay. Under light irradiation (760 nm, 500 mW cm^−2^, 10 min), green fluorescence (live cells) gradually diminished with increasing concentrations of ER‐BDP NPs, while red fluorescence (dead cells) intensified accordingly. Notably, at 20 μM, green fluorescence completely disappeared, indicating complete tumor cell eradication (Figure 3d).

To validate the light‐dependent nature of the cytotoxic effect, 4T1 cells were divided into four groups: PBS, PBS + light, ER‐BDP NPs alone, and ER‐BDP NPs + light. Live/dead staining revealed that cells in the first three groups displayed predominantly green fluorescence, indicating no significant cell death. In contrast, cells in ER‐BDP NPs + light exhibited strong red fluorescence, confirming extensive cell death. These results demonstrate that significant cytotoxicity is induced only upon exposure to light irradiation in the presence of ER‐BDP NPs, whereas no apparent cell damage occurs under dark conditions (Figure 3e).

### 1.5 In vivo antitumor efficacy and biosafety evaluation

**FIGURE 4:**
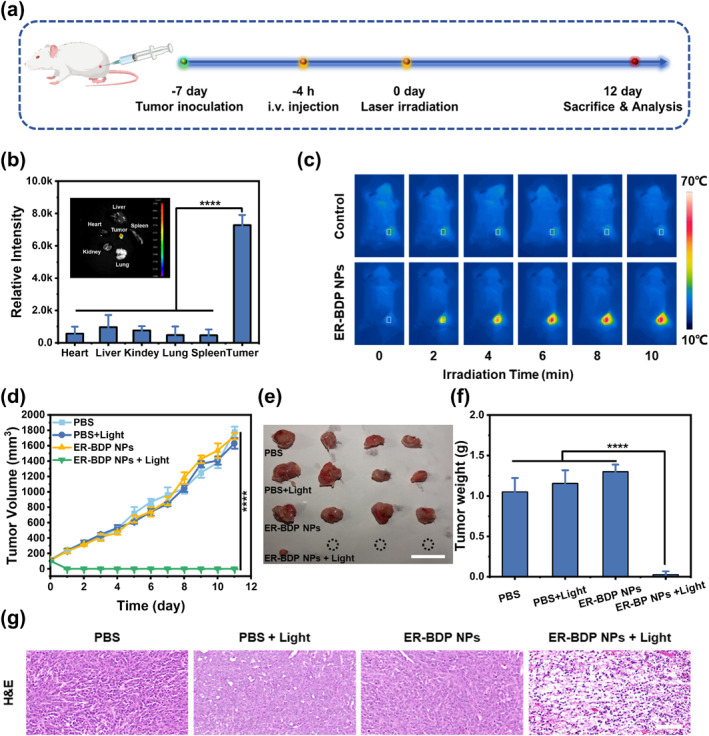
In vivo antitumor evaluation based on the subcutaneous tumor model. (a) Schematic illustration of the establishment of the 4T1 subcutaneous tumor mouse model and the administration protocol. (b) Fluorescence distribution intensity of ER‐BDP NPs in various organs at 6 h post tail vein injection and quantitative analysis of fluorescence distribution of ER‐BDP NPs in major organs and tumors. (c) Thermal infrared images of 4T1 tumor‐bearing mice at 6 h post intravenous injection of ER‐BDP NPs or normal saline followed by 10 min laser irradiation. (d) Tumor volume change curves of mice in different treatment groups within 12 days (*n* = 4). (e) Photographs of excised tumors from the subcutaneous tumor model after different treatments on day 12. Scale bar: 2 cm. (f) Tumor weights of mice in different treatment groups (*n* = 4). (g) H&E staining images of tumor tissues from different treatment groups. Scale bar: 200 μm. Statistical significance was calculated by one‐way ANOVA with Tukey's post hoc test. ER‐BDP NPs, endoplasmic reticulum‐targeting BODIPY‐based photothermal agent nanoparticles. *p < 0.05, **p < 0.01, ***p < 0.001, ****p < 0.0001.

Encouraged by the promising in vitro therapeutic performance of the ER‐BDP NPs, we further evaluated their in vivo PTT efficacy using 4T1 tumor‐bearing BALB/c mice (Figure 4a). The breast tumor model was established by subcutaneously inoculating 4T1 cells 1 week prior to treatment. Subsequent experiments were conducted when the tumors reached a mean volume of approximately 100 mm^3^.

To determine the optimal irradiation time, we first evaluated the tumor accumulation of ER‐BDP NPs in tumor‐bearing mice. Following intravenous injection of ER‐BDP NPs, in vivo fluorescence imaging was performed using a small‐animal imaging system to monitor their biodistribution. The results showed that the fluorescence signal at the tumor site increased over time and peaked at 6 h post‐injection, demonstrating maximum tumor uptake at this time point (Supporting Information S1: Figure S5).

When the accumulation of ER‐BDP NPs in the tumor reached its maximum, the mice were euthanized, and the tumors along with major organs were excised and imaged ex vivo using a small‐animal imaging system. Fluorescence intensity analysis revealed that the vast majority of ER‐BDP NPs were retained in the tumor region with a significantly higher signal compared to that in the heart, liver, spleen, lungs, and kidney (Figure 4b). These results indicate that ER‐BDP NPs can achieve specific targeting and effective enrichment in tumor tissues. This excellent targeting and accumulation profile enables ER‐BDP NPs to exert tumor photothermal ablation while maintaining good biocompatibility toward other organs and tissues.

To evaluate the in vivo photothermal antitumor efficacy of ER‐BDP NPs, the four groups of mice were administered the corresponding agents. At 4 h post‐injection, tumors were either irradiated with a 760 nm laser (500 mW cm^−2^, 10 min) or kept in the dark. During irradiation, infrared thermal imaging showed a marked temperature increase in the tumors of mice injected with ER‐BDP NPs, whereas no significant temperature change was observed in tumors of mice receiving PBS only (Figure 4c and Supporting Information S1: Figure S6). This confirms that ER‐BDP NPs maintain efficient photothermal conversion in vivo.

Importantly, clear differences were observed in tumor growth. Tumors in PBS, PBS + Light and ER‐BDP NPs treatment groups exhibited rapid growth, with volume increases of 15.1‐fold (PBS group), 16.2‐fold (PBS + Light group), and 15.8‐fold (ER‐BDP NPs group) over 12 days. Conversely, tumor growth in the ER‐BDP NPs + Light treatment group was strongly suppressed, with tumor volume decreasing from day 2 post‐treatment (Figure 4d). Consistent with this, excised tumors from the ER‐BDP NPs + Light treatment group showed markedly reduced size and weight compared with those from the other groups (Figure 4e,f). Marked tumor regression was observed in the ER‐BDP NPs + Light treatment group as early as day 2 post‐treatment, and no evident tumor recurrence occurred throughout the subsequent observation period. These findings are in good agreement with the quantitative data shown in the figures.

To further examine tumor cell damage following ER‐BDP NP treatment, paraffin sections of tumor tissues were stained with hematoxylin and eosin (H&E). Histological analysis revealed severe tissue damage in tumors from the ER‐BDP NPs + Light treatment group, whereas tumors from the other groups remained largely unaffected (Figure 4g). These results demonstrate that ER‐BDP NPs serve as potent photothermal agents that efficiently induce tumor cell death, underscoring a precision‐guided strategy based on organelle‐specific targeting.

To investigate potential effects on normal organs and tissues during in vivo treatment, in addition to monitoring body weight and general locomotor activity, we performed hematoxylin and eosin (H&E) staining on major organs (heart, liver, spleen, lungs, and kidney) from each group to assess the biosafety of ER‐BDP NPs. Concurrently, routine blood tests were conducted. The results showed no obvious organ damage or metastatic lesions in any group, and H&E staining revealed no marked histopathological abnormalities (Supporting Information S1: Figure S7). Blood parameters including white blood cell (WBC) count, red blood cell (RBC) count, hemoglobin (HGB) concentration, mean corpuscular volume (MCV), platelet (PLT) count, and hematocrit (HCT), all fell within normal ranges with no significant deviations (Supporting Information S1: Figure S8). These findings indicate that the treatment did not induce notable organ injury or systemic physiological disturbances, confirming that ER‐BDP NPs possess excellent biocompatibility toward normal tissues and organs while preserving potent antitumor efficacy in vivo. Body weight measurements showed only a mild increase in all groups without significant inter‐group differences (Supporting Information S1: Figure S9), indicating that ER‐BDP NPs did not induce notable systemic stress.

## 2 CONCLUSION

In summary, we have developed a novel ER‐targeting photothermal agent, ER‐BDP, by integrating a trifluoromethyl‐substituted BODIPY core with a p‐toluenesulfonyl‐based ER‐targeting moiety. ER‐BDP NPs, formulated via co‐assembly with DSPE‐PEG_2000_‐Biotin, exhibited high photothermal conversion efficiency (80.3%) and superior organelle‐level precision. Systemic evaluations demonstrated that ER‐BDP NPs could achieve efficient lysosomal escape and near‐complete tumor ablation at low dosages with minimal systemic toxicity. This work not only provides an effective nanoplatform for precise cancer therapy but also offers a valuable molecular design strategy for developing next‐generation organelle‐specific functional dyes.

## CONFLICT OF INTEREST STATEMENT

The authors declare no conflicts of interest.

## ETHICS STATEMENT

The animal experiments were approved by the Animal Ethics Committee of the Dalian University of Technology (DUT20230822).

## Supporting information

Supporting Information S1

## Data Availability

The data that support the findings of this study are available in the Supporting Information of this article.
