# Supplementary material for: A lysosomal escape‐enabled endoplasmic reticulum‐targeting BODIPY photothermal agent for enhanced tumor ablation
Source: Smart Mol. 2026 Jun 24:e70073. Online ahead of print. doi: 10.1002/smo2.70073 (PMC13399612; doi:10.1002/smo2.70073)
Supplement: Supplementary file 1 — Supporting Information S1 [file SMO2-9999-0-s001.docx]

Supporting Information

**A Lysosomal Escape-Enabled Endoplasmic Reticulum-Targeting BODIPY Photothermal Agent for Enhanced Tumor Ablation**

Shaoyang Shi^1^, Yanbing Cao^1^ *, Jiexuan Zuo^1^, Qiushi Li^1^, Xiaolong Zeng^1^, Jianjun Du^1^, Jiangli Fan^1^, Wen Sun^1,2^ *, Xiaojun Peng^1^

^1^ State Key Laboratory of Fine Chemicals, Frontiers Science Center for Smart Materials Oriented Chemical Engineering, Dalian University of Technology, Dalian 116024, China.

^2^ Ningbo Institute of Dalian University of Technology, Ningbo 315016, China.

E-mail: yanbingcao@dlut.edu.cn; sunwen@dlut.edu.cn)

**Keywords:** BODIPY, Photothermal therapy, Lysosomal escape, Endoplasmic reticulum targeting, Nanomedicine

**1. Materials**

All reagents and chemicals utilized in this study were procured from Bide Pharmatech Co., Ltd (China) and were employed without additional purification. All the other solvents and reagents used in this study were of analytical grade. The chemical reagents employed in this study were obtained from Energy-Chemical or Bidepharm. All standard reagents for fluorescence assays, including MTT (3-(4,5-dimethyl-2-thiazolyl)-2,5-diphenyl-2-H-tetrazolium bromide), were acquired from Energy Chemical Co. Lyso Tracker Green, ER Tracker Green, annexin V-FITC/propidium iodide (PI) apoptosis detection kit, and calcein-AM/propidium iodide (PI) detection kit were all purchased from Beyotime Biotechnology Co. (China). Unless noted otherwise, all additional reagents were commercially sourced and utilized without further modification. Murine breast cancer 4T1 cells, human non-small cell lung cancer A549 cells, and human breast cancer MCF-7 cells were all acquired from the Institute of Basic Medical Sciences (IBMS) of the Chinese Academy of Medical Sciences.

**2. Methods**

^1^H-NMR and ^13^C-NMR spectra were recorded on a Bruker Avance III 400 spectrometer. Mass spectrometry (MS) data were obtained using an LTQ Orbitrap XL system. Absorption spectra were measured using a UV-Vis spectrophotometer (Lambda 750S). The diameter of nanoparticles was determined by dynamic light scattering (DLS) on a Malvern Zetasizer Nano ZS90 (Malvern, UK). Both in vitro and in vivo experiments utilized a laser with λ = 760 nm as the light source. Laser power output was regulated by a fiber-coupled laser system (FCW-760-30W, Changchun New Industries Optoelectronics Tech Co., Ltd.) and quantified using a power meter (CEL-NP2000, Beijing Zhongjiao Jinyuan Tech Co., Ltd.). Confocal laser scanning microscopy (CLSM) images were captured using an Olympus FV3000 confocal laser scanning microscope.

The animal experiments were approved by the Animal Ethics Committee of the Dalian University of Technology (DUT20230822). Data were obtained from at least three independent measurements (n ≥ 3) and presented as mean ± standard deviation (SD).

**3. Synthesis**


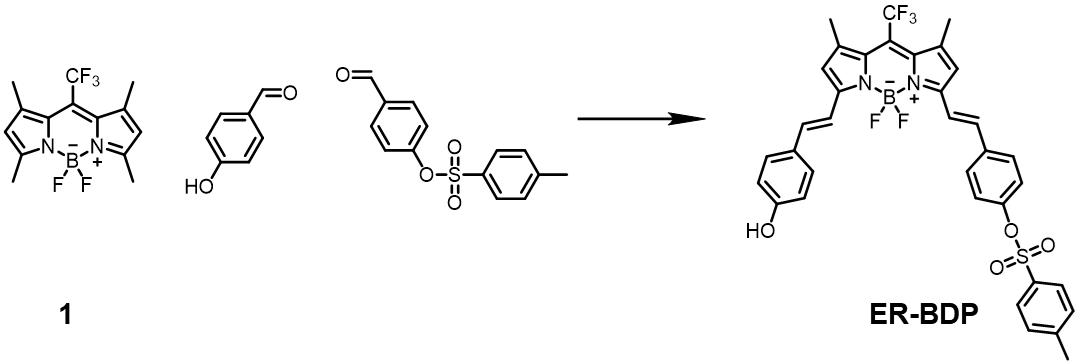


**Scheme S1.** Synthesis Routes of the ER-BDP

**Synthesis of Compound 1:** Compound 1 was synthesized according to the reported literature.^[1]^

^1^H NMR (400 MHz, CDCl_3_) δ 6.15 (s, 1H), 2.54 (s, 3H), 2.30 (s, 3H). MS (APCI)：[M+H]^+^ calcd for C_35_H_27_BF_5_N_2_O_4_S: 316.1, found: 317.1.

**Synthesis of ER-BDP：**Under a nitrogen atmosphere, Compound 1 (1g, 2.81 mmol), *p*-hydroxybenzaldehyde (386 mg, 2.81 mmol), and 4-[[(4-methylphenyl)sulfonyl]oxy]benzalehyde (874 mg, 2.80 mmol), acetic acid (0.1 mL), and piperidine (0.1 mL) were added to dry toluene (10 mL), with a small amount of activated 4Å molecular sieves, and the mixture was refluxed and stirred at 100 °C for 3 hours. Once cooled to room temperature, the reaction mixture was extracted with DCM. The combined organic phases were washed with water, dried with anhydrous Na_2_SO_4_, and evaporated under reduced pressure. The crude product was purified by silica gel column chromatography (DCM/MeOH 50:1) to yield Compound ER-BDP (1.87g, 87%). ^1^H NMR (400 MHz, DMSO) δ 7.76 (d, *J* = 7.8 Hz, 3H), 7.58 (d, *J* = 17.1 Hz, 5H), 7.49 (d, *J* = 8.0 Hz, 3H), 7.36 (d, *J* = 20.2 Hz, 2H), 7.10 (d, *J* = 8.1 Hz, 3H), 6.87 (d, *J* = 8.0 Hz, 2H), 2.43 (s, 3H), 2.32 (s, 6H). HRMS (ESI)：[M-H]^+^ calcd for C_35_H_27_BF_5_N_2_O_4_S: 677.1710, found: 677.1700.

**4. Sample Preparation**

1 mg of ER-BDP and 10 mg of DSPE-PEG_2000_-Biotin were dissolved in 1 mL of THF. Under 300 W ultrasonication, the above THF solution was added dropwise to 9 mL of ultrapure water, and ultrasonication was continued for 5 minutes until a visually clear and stable nanodispersion was formed. The preliminary nanoparticle solution was successively filtered 0.45 μm aqueous membranes to yield a nanoparticle solution with more uniform particle sizes. Subsequently, the filtered nanoparticle solution was dialyzed overnight in a 3500 Da molecular weight cutoff dialysis bag to eliminate organic solvents, producing the final aqueous nanoparticle solution (ER-BDP NPs).

The nanoparticles were demulsified in DMSO, and the resulting solution was subjected to UV-Vis absorption spectroscopy. The concentration of the original nanoparticle dispersion was then determined by fitting the obtained absorbance values to the calibration curve shown in Fig. S1.

**5. Photothermal conversion efficiency calculation**

The photothermal conversion efficiency (η) of B@Q was calculated using the following equation:

$$\eta=\frac{hs({\Delta T}_{ER-BDP NPs}-{\Delta T}_{water})}{I(1-{10}^{A_{760}})}$$

h is the heat transfer coefficient; s is the surface area of the container; ∆T_B@Q_ represents the temperature rise of the ER-BDP NPs aqueous solution; ∆T_water_ represents the temperature rise of pure water; I is the laser power, and A is the absorbance at 760 nm wavelength.

$$hs=\frac{mc_{water}}{\tau_{s}}$$

m is the mass of the B@Q aqueous solution system; c_water_ is the specific heat capacity of the solution (c_water_ = 4.2 J/(g·°C)); τ_s_ is the associated time constant.

$$t=-\tau_{s}\ln\left( \theta\right)$$

θ is a dimensionless parameter known as the driving temperature.

$$\theta=\frac{T-T_{Surr}}{T_{Max}-T_{Surr}}$$

T_Max_ is the maximum steady-state temperature; T_Surr_ is the ambient temperature.

**6. Cell experiments**

**6.1 Cell incubation**

4T1 mouse breast cancer cells were cultured in DMEM medium containing 10% fetal bovine serum (FBS), 1% penicillin and streptomycin, under an atmosphere of 5% CO_2_ and 95% humidified air. Through regular subculturing, the cells were maintained in the exponential growth phase. Prior to experiments, cell density was determined with a cell counter.

A549 cells were cultured in DMEM medium supplemented with 10% FBS and 1% penicillin‑streptomycin under a humidified atmosphere containing 5% CO₂ at 37 °C. Cells were routinely subcultured to maintain exponential growth, and cell density was determined using a cell counter prior to each experiment.

MCF-7 human breast cancer cells were cultured in DMEM medium supplemented with 10% FBS and 1% penicillin-streptomycin, under an atmosphere of 5% CO₂ and 95% humidified air. Cells were maintained in the exponential growth phase through regular subculturing. Prior to experiments, cell density was determined using a cell counter.

**6.2 Cellular uptake and organelle co-localization**

4T1 cells were seeded at a density of 1×10⁵ cells/mL in 35 mm confocal dishes and cultured overnight. The cells were co-incubated with 10 μM ER-BDP NPs, and at regular intervals, fluorescence imaging of ER-BDP NPs was performed using a confocal microscope (Olympus FV-3000 with a 60 × objective lens) to investigate the cellular uptake of ER-BDP NPs.

The cells were co-incubated with 10 μM ER-BDP NPs and Lyso Tracker Green, ER Tracker Green were prepared according to the manufacturer’s instructions, and at regular intervals, fluorescence imaging of ER-BDP was performed using a confocal microscope (Olympus FV-3000 with a 60× objective lens) to investigate the subcellular organelle colocalization of ER-BDP NPs.

**6.3 Dead/Live cell co-staining**

4T1 cells were plated in confocal dishes and incubated for 24 hours. Once the desired cell density was achieved, cells were co-cultured with 0-20 μM ER-BDP NPs for 4 hours or 20 μM on different treatments (G1-G4: PBS, PBS+ Light, ER-BDP NPs, ER-BDP NPs+ Light). The cells were irradiated with a laser (760 nm, 500 mW cm⁻²) for 10 minutes or continued to be cultured in the dark. After further culturing for 12 hours, the cells were stained with Calcein-AM and propidium iodide. The excitation wavelength was 488 nm, with the green channel emission wavelength at 505-545 nm and the red channel emission wavelength at 600-700 nm.

**6.4 Cytotoxicity experiments**

4T1, MCF-7, and A549 cells were cultured in 96-well plates (approximately 1×10⁴ cells per well, in 100 μL of culture medium). After 24 hours of cell incubation, different concentrations of ER-BDP NPs were co-incubated with the cells for 4 hours. These cells were exposed to a 500 mW cm⁻², 760 nm laser for 10 minutes. Simultaneously, under the same experimental conditions, cells co-incubated with ER-BDP NPs but not exposed to light were tested for dark toxicity. Following an additional 24 hours of incubation, MTT solution (100 μL, 0.5 mg/mL, DMEM) was introduced into each well. After continued incubation at 37 °C for 4 hours, the absorbance of each well at 490 nm was measured using a microplate reader to calculate cell viability:

$$C\mathrm{ell} \mathrm{viability} \left( \% \right)=\frac{{OD}_{ps}-{OD}_{blank}}{{OD}_{control}-{OD}_{blank}}\times100\%$$

OD denotes optical density (absorbance).

**7 *In vivo* experiments**

**7.1 Animals and tumor model**

This study was conducted in accordance with the Guide for the Care and Use of Laboratory Animals published by the US National Institutes of Health (8th edition, 2011). The female BALB/c mice aged about 4-5 weeks were purchased from Liaoning Changsheng biotechnology Co. Ltd. The animal protocol was approved by the local research ethics review board of the Animal Ethics Committee of the Dalian University of Technology (DUT20230822). The subcutaneous tumor model was established by subcutaneously injecting 50 μL of 5 × 10^6^ 4T1 cells suspended in PBS into the right hind leg of the mouse. Tumors that reached an approximate volume of 100 mm3 were deemed suitable for experimental use. The tumor volume (V) of 4T1 tumor-bearing mice was calculated using the formula:

$$V=\frac{ab^{2}}{2}$$

a: mouse tumor length; b: mouse tumor width.

When the tumor volume reached about 100-150 mm³, the mice were utilized for *in vivo* imaging and photothermal therapy (PTT).

The mice were randomly divided into 4 groups: 1) the PBS group of mice administrated with PBS (100 μL) alone; 2) the PBS + Light group of mice administrated with PBS and irradiated with 760 nm irradiation (500 mW cm^-2^) for 10 min; 3) the group of mice administrated with ER-BDP NPs (1 mg mL^-1^, 100 μL) alone; 4) the ER-BDP NPs + Light group of mice administrated with ER-BDP NPs and irradiated with 760 nm irradiation (100 mW cm^−2^) for 10 min. The light irradiation was performed after 6 h injection after the injection of PBS or ER-BDP NPs. The tumor growth and body weight change were monitored every 2 days. The tumors and major organs (e.g., hearts, livers, lungs, spleens and kidneys) were harvested at the end of antitumor studies for immunofluorescence examination and H&E staining.

**7.2 *In vivo* fluorescence imaging**

The distribution of ER-BDP NPs in 4T1 tumor-bearing mice was evaluated using fluorescence signals. 100 µL of ER-BDP NPs (40 μM) was injected via the tail vein. Under general anesthesia, fluorescence imaging of the mice was performed at different time points using an *in vivo* imaging system (IVIS Lumina imaging system).

**8.Statistical analysis**

Data analyses were conducted using the GraphPad Prism 8 software. For variance analysis, One-way analysis of variance (ANOVA) with Tukey’s post hoc test was used. p values of <0.05 were considered significant. *p < 0.05, **p < 0.01, ***p < 0.001, and ****P < 0.0001.


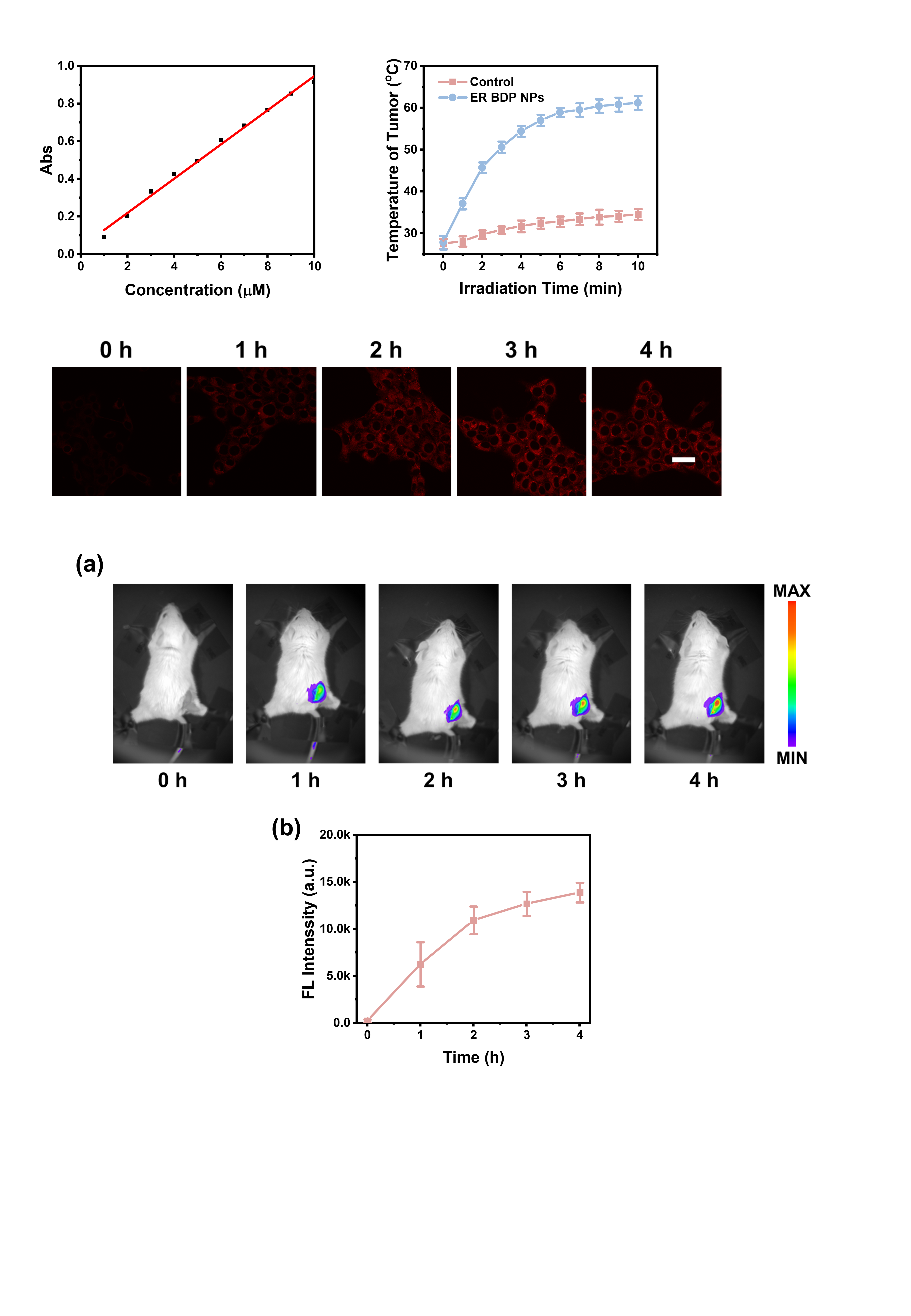


**Fig. S1.** Absorption spectra of ER-BDP NPs at different concentrations in DMSO.


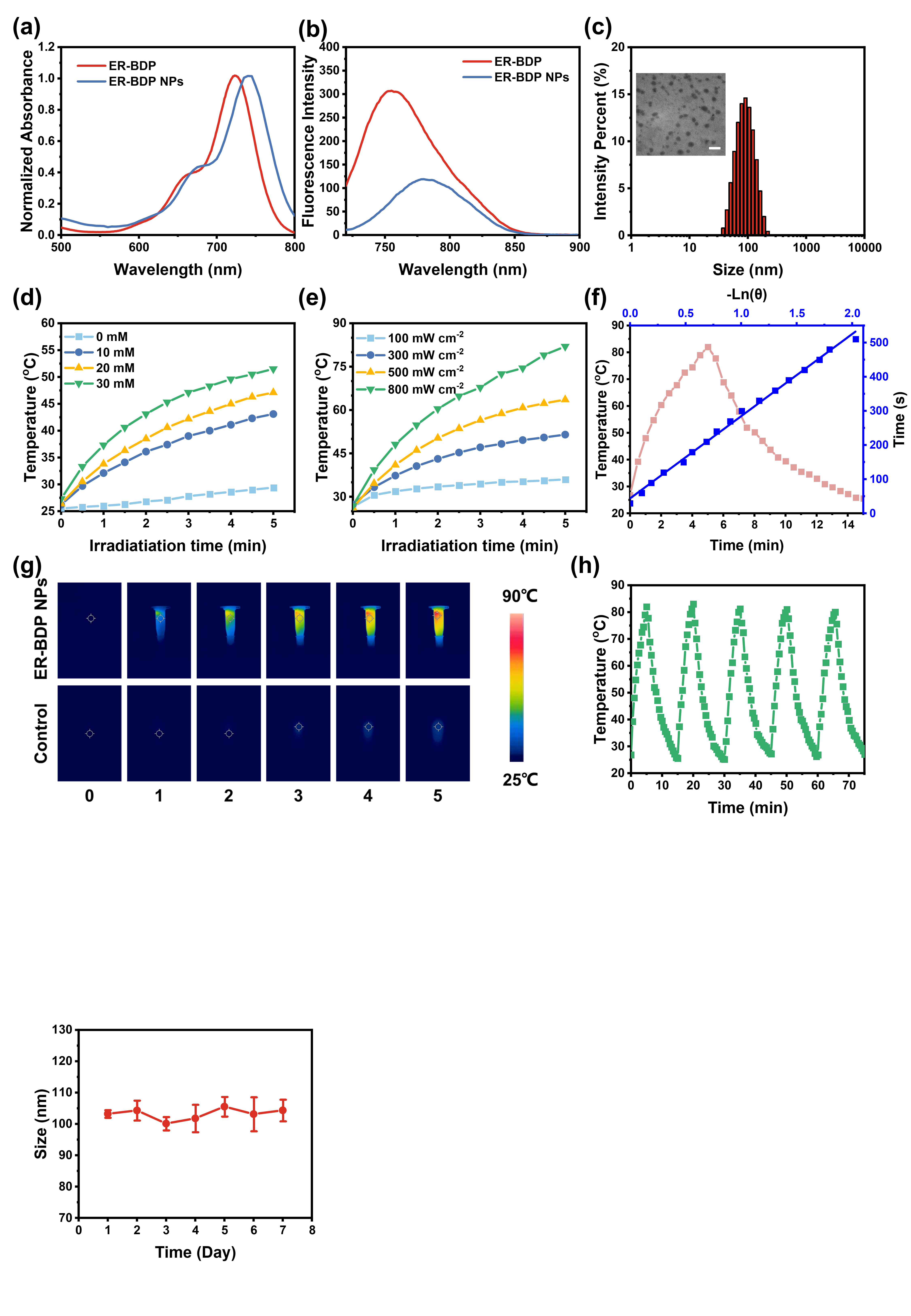


**Fig. S2.** Variations in the hydrodynamic diameter of ER-BDP NPs in aqueous solution.


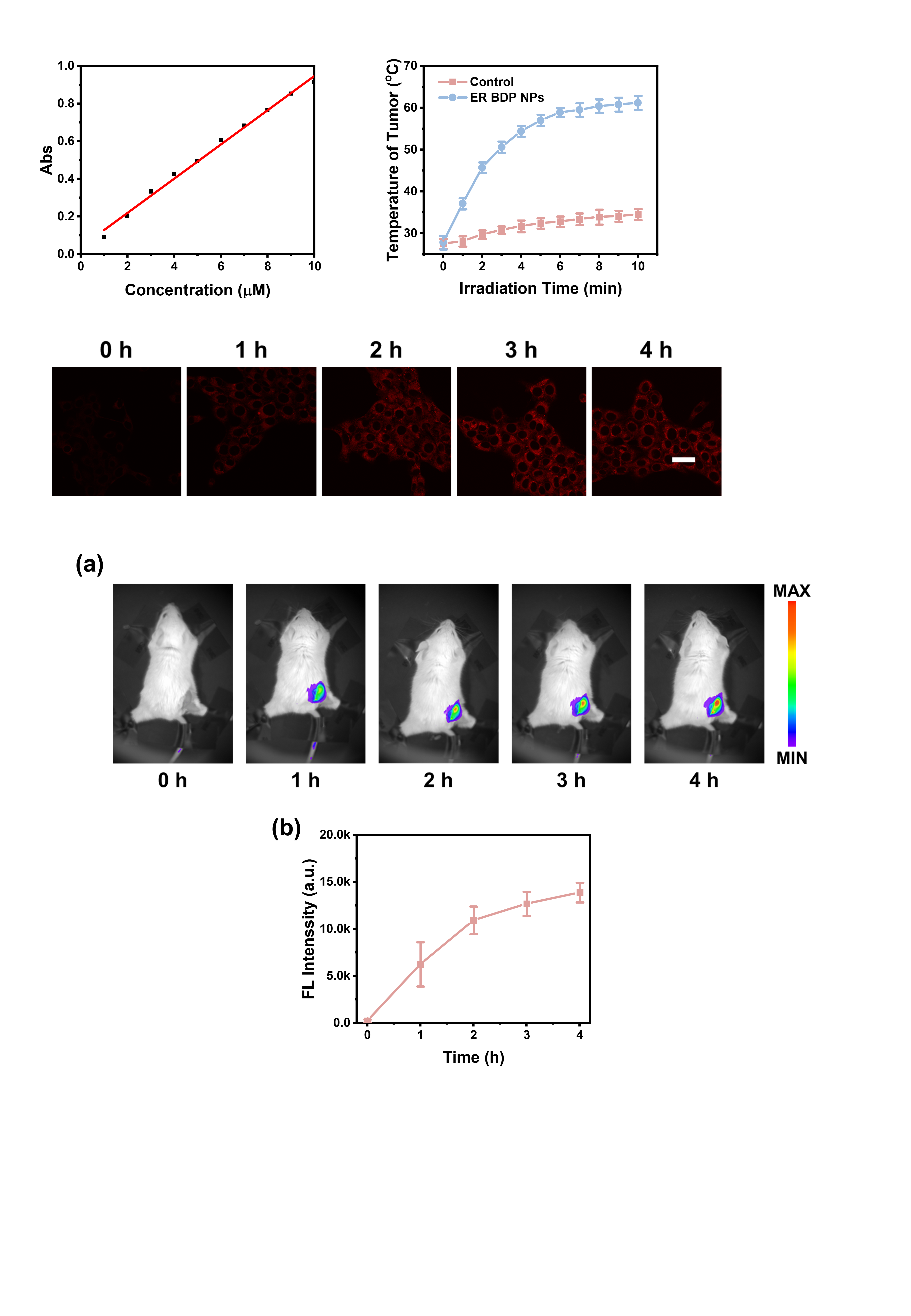


**Fig. S3.** Confocal fluorescence microscopic images showing the cellular internalization process of dye-loaded ER-BDP NPs in 4T1 cells over time. Scale bar: 50 μm.


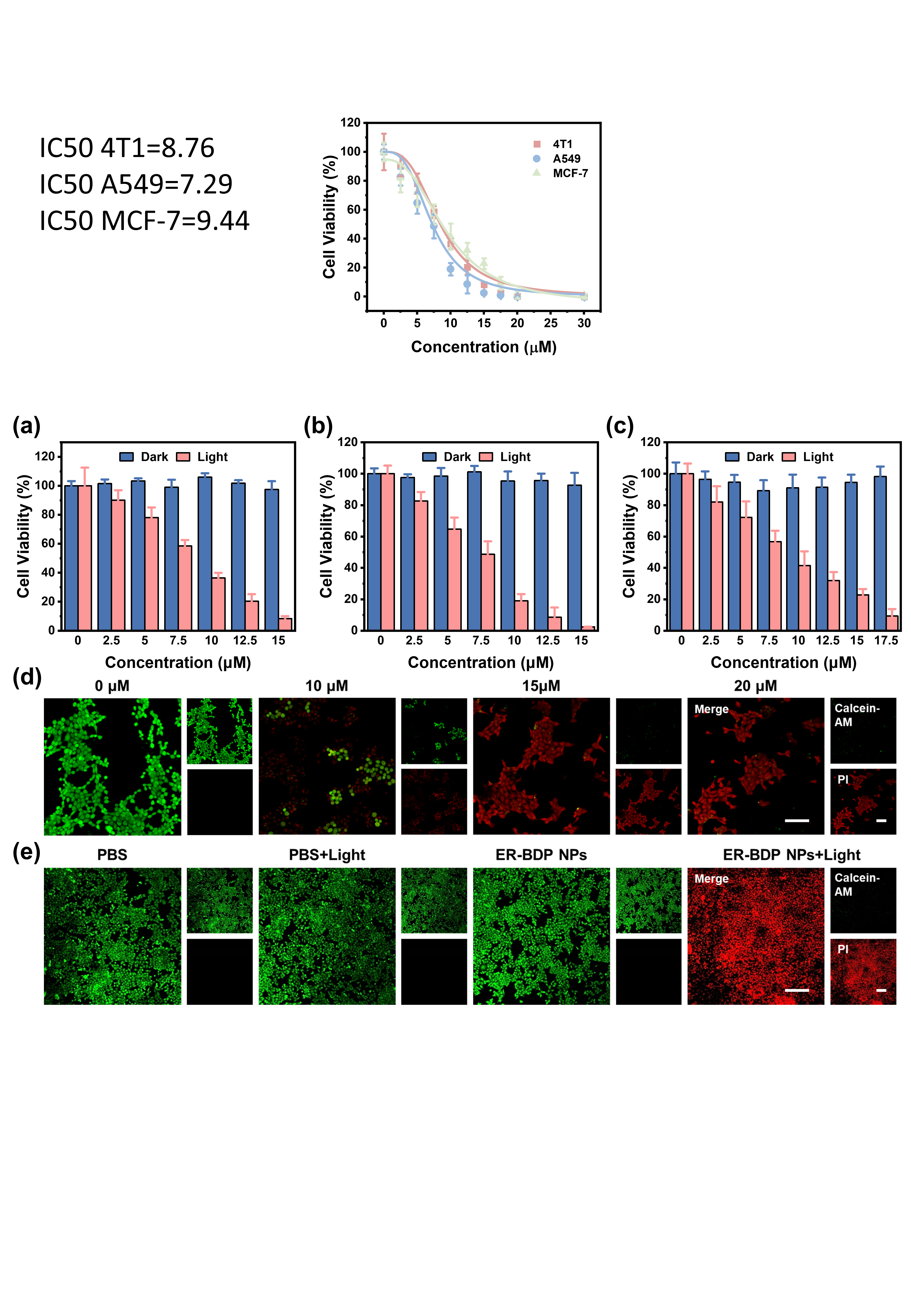


**Fig. S4.** The half‑maximal inhibitory concentration (IC50) of ER‑BDP NPs under laser irradiation after incubation with 4T1, A549, and MCF‑7 cells.


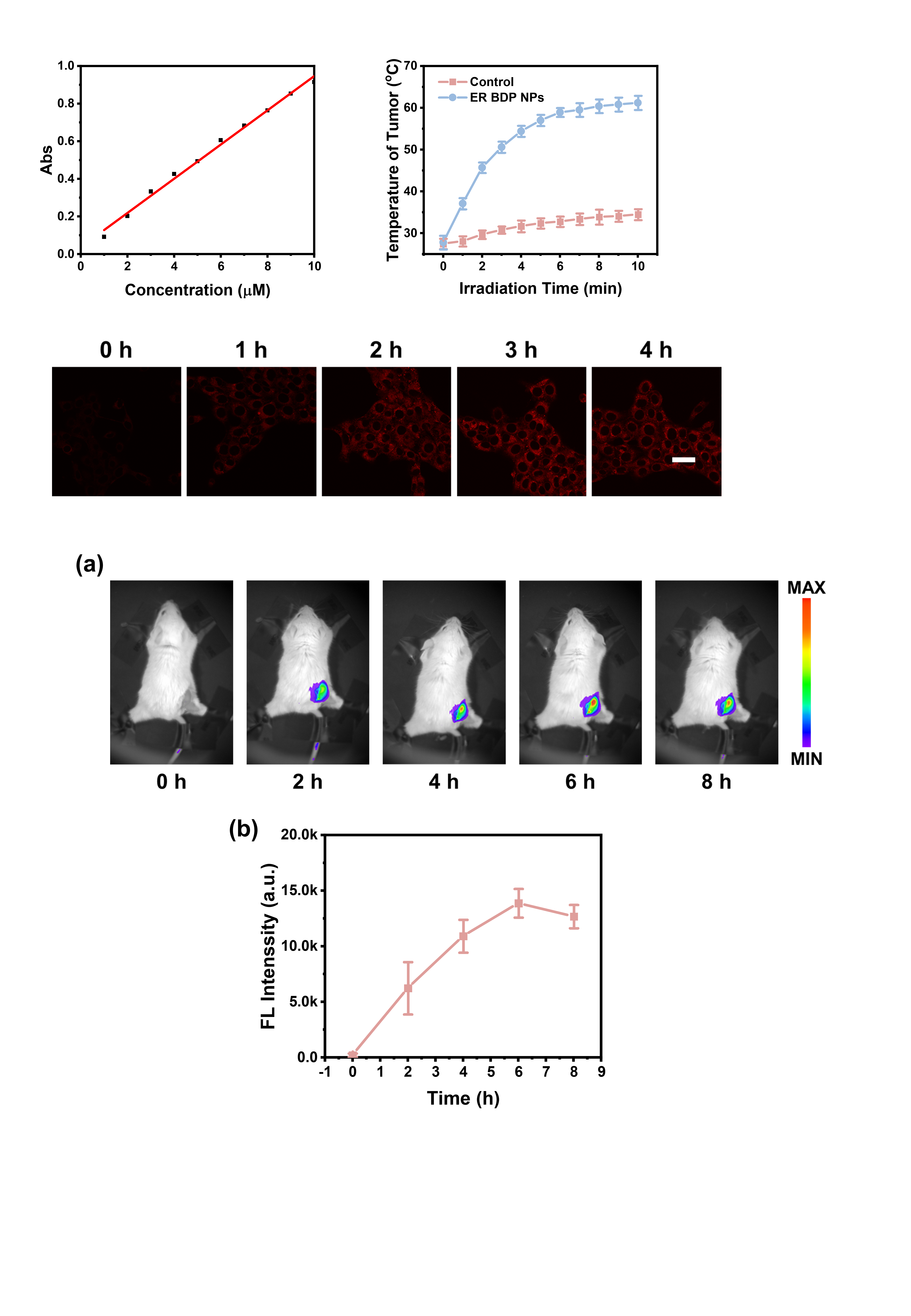


**Fig. S5.** Fluorescence intensity distribution images of ER-BDP NPs at the tumor site over different time points(a) and corresponding quantitative analysis(b).


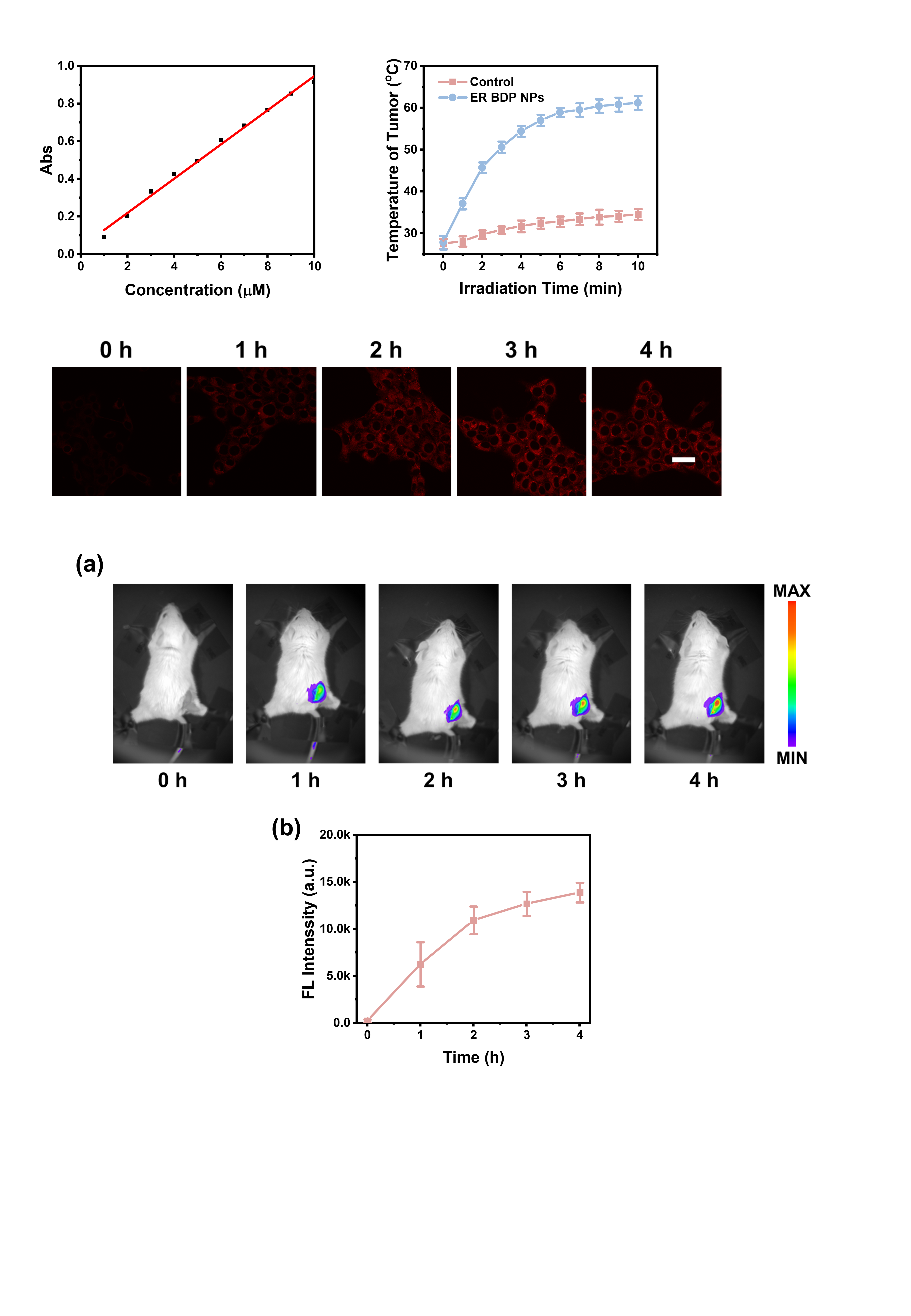


**Fig. S6.** Temperature change curves of 4T1 tumors in mice injected with ER-BDP NPs or normal saline during laser irradiation.

**Fig. S7.** HE-Stained sections of major organs from mice in different groups. Scale bar = 200 μm


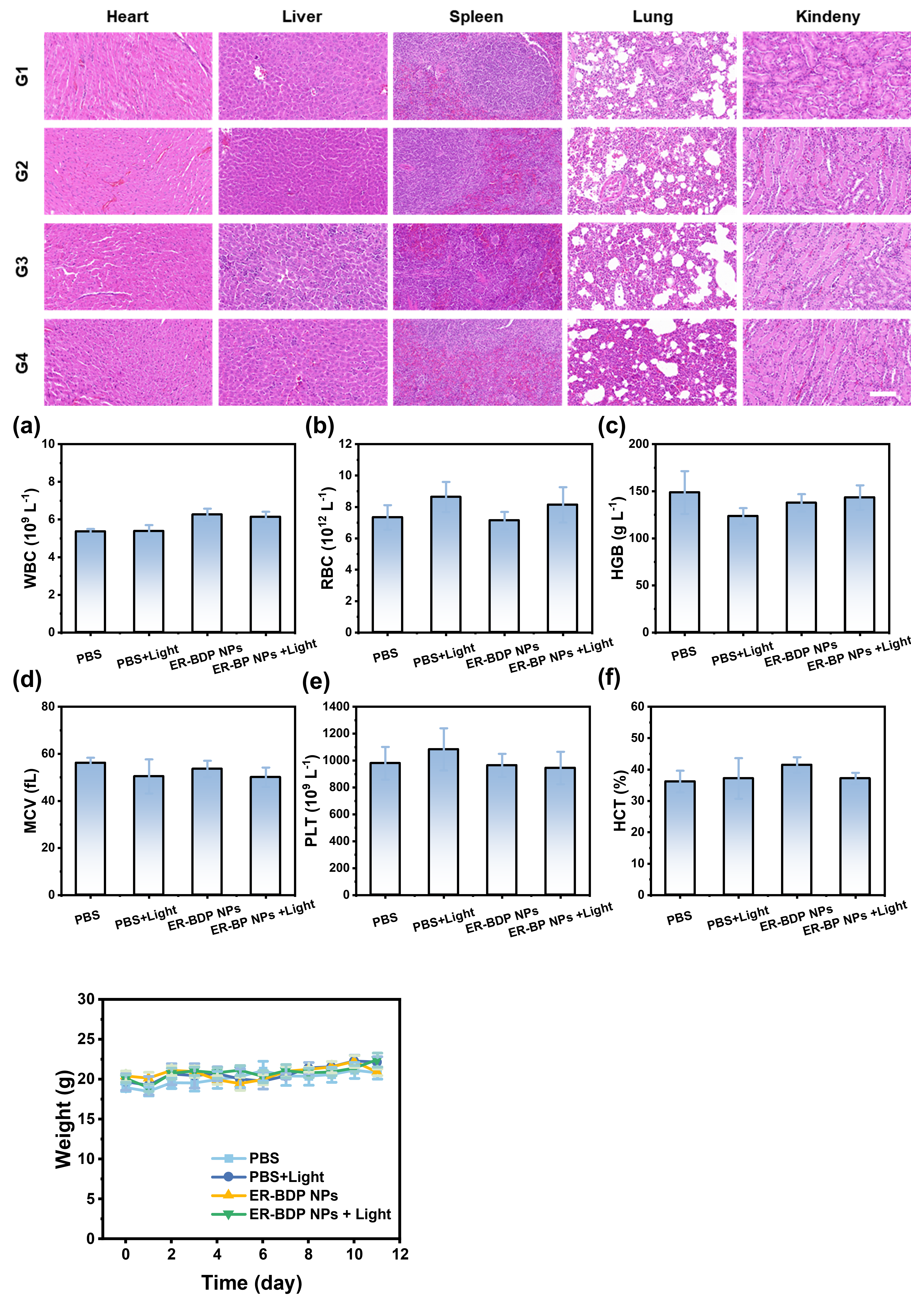


**Fig. S8.** Blood parameters including white blood cell (WBC) count (a), red blood cell (RBC) count (b), hemoglobin (HGB) concentration (c), mean corpuscular volume (MCV) (d), platelet (PLT) count (e), and hematocrit (HCT) (f)


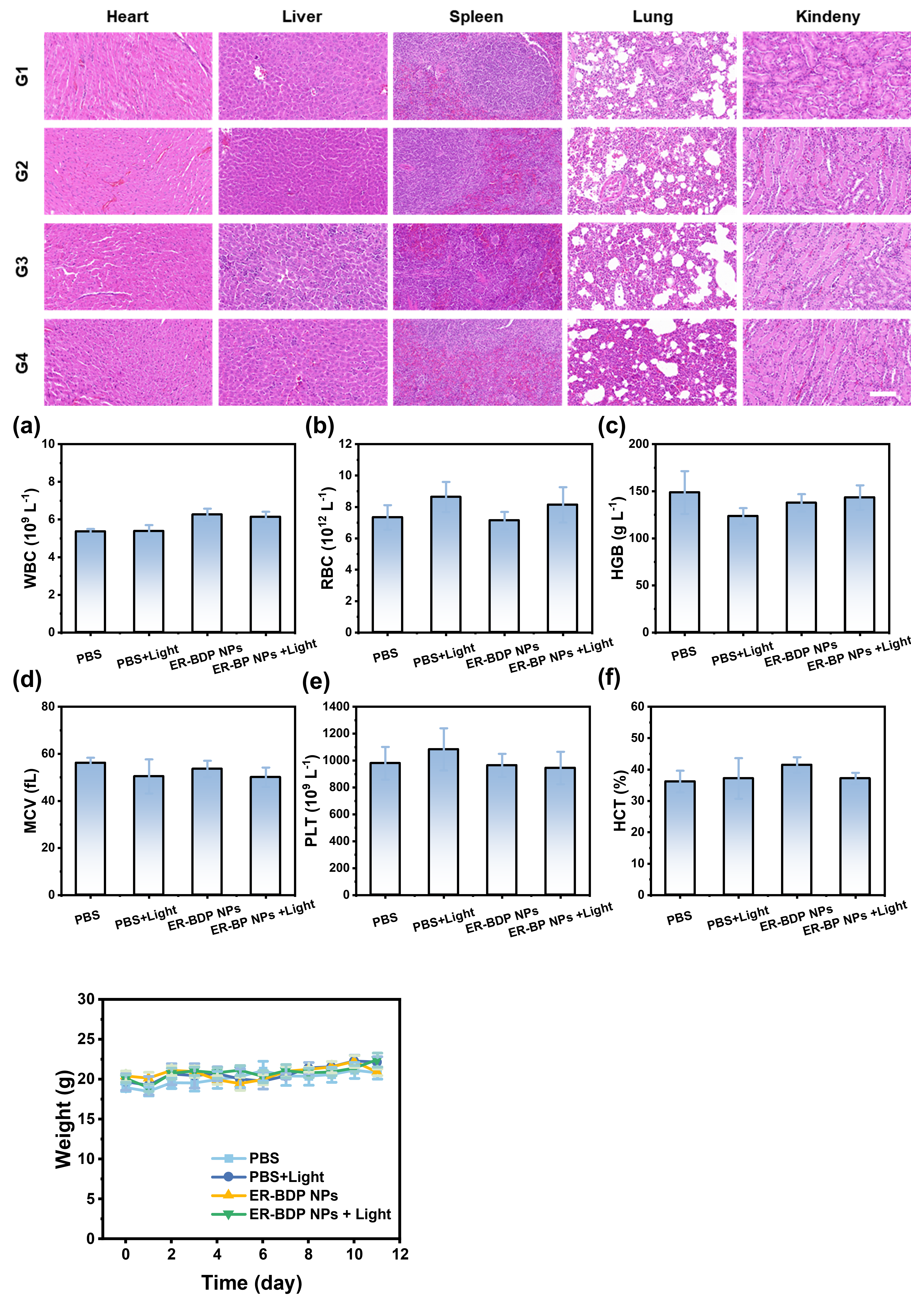


**Fig. S9.** Body weight change curves of mice in different treatment groups (n=5).


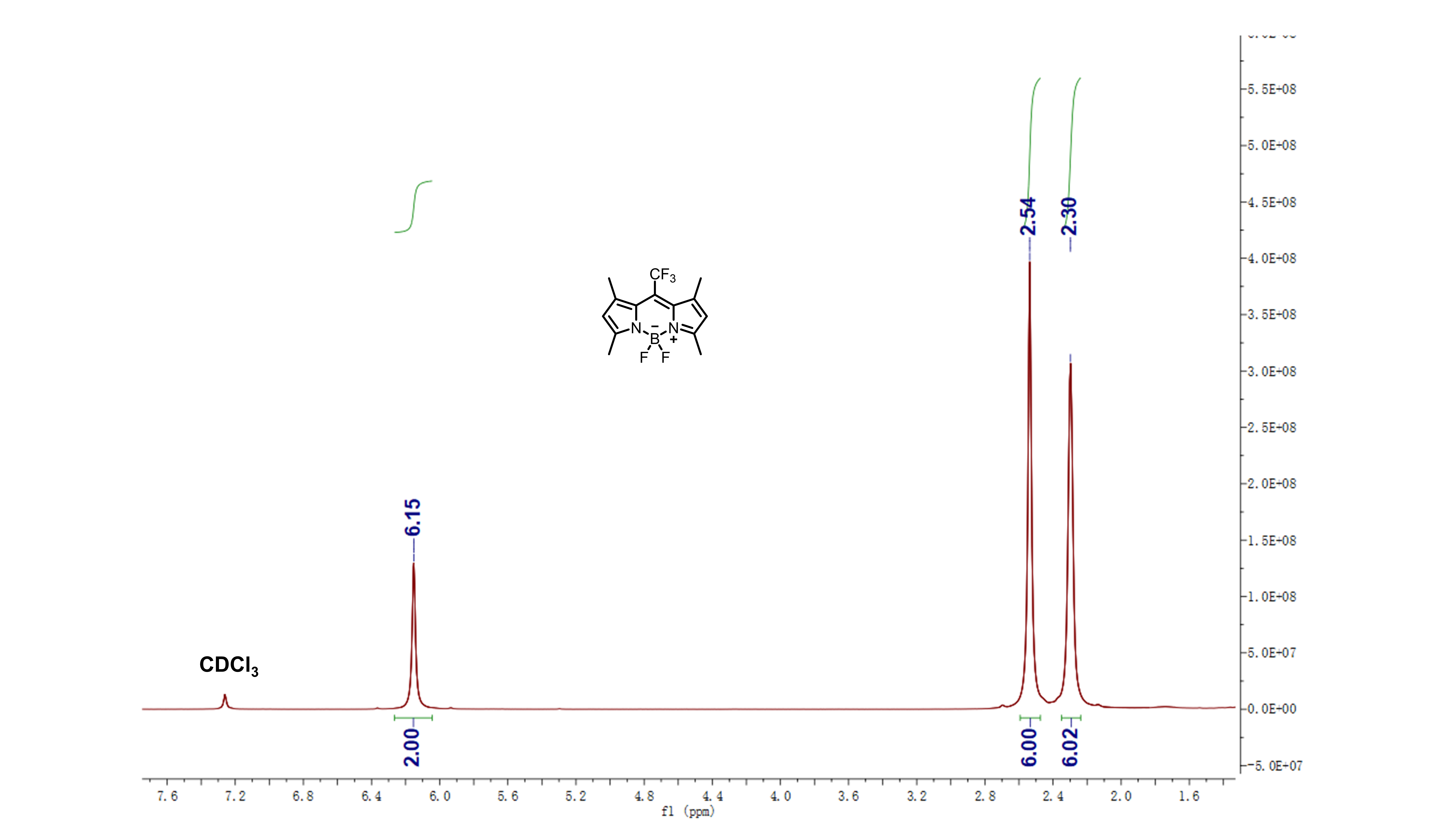


**Fig. S10** ^1^H NMR spectrum of Compound 1 (400 MHz, CDCl_3_).


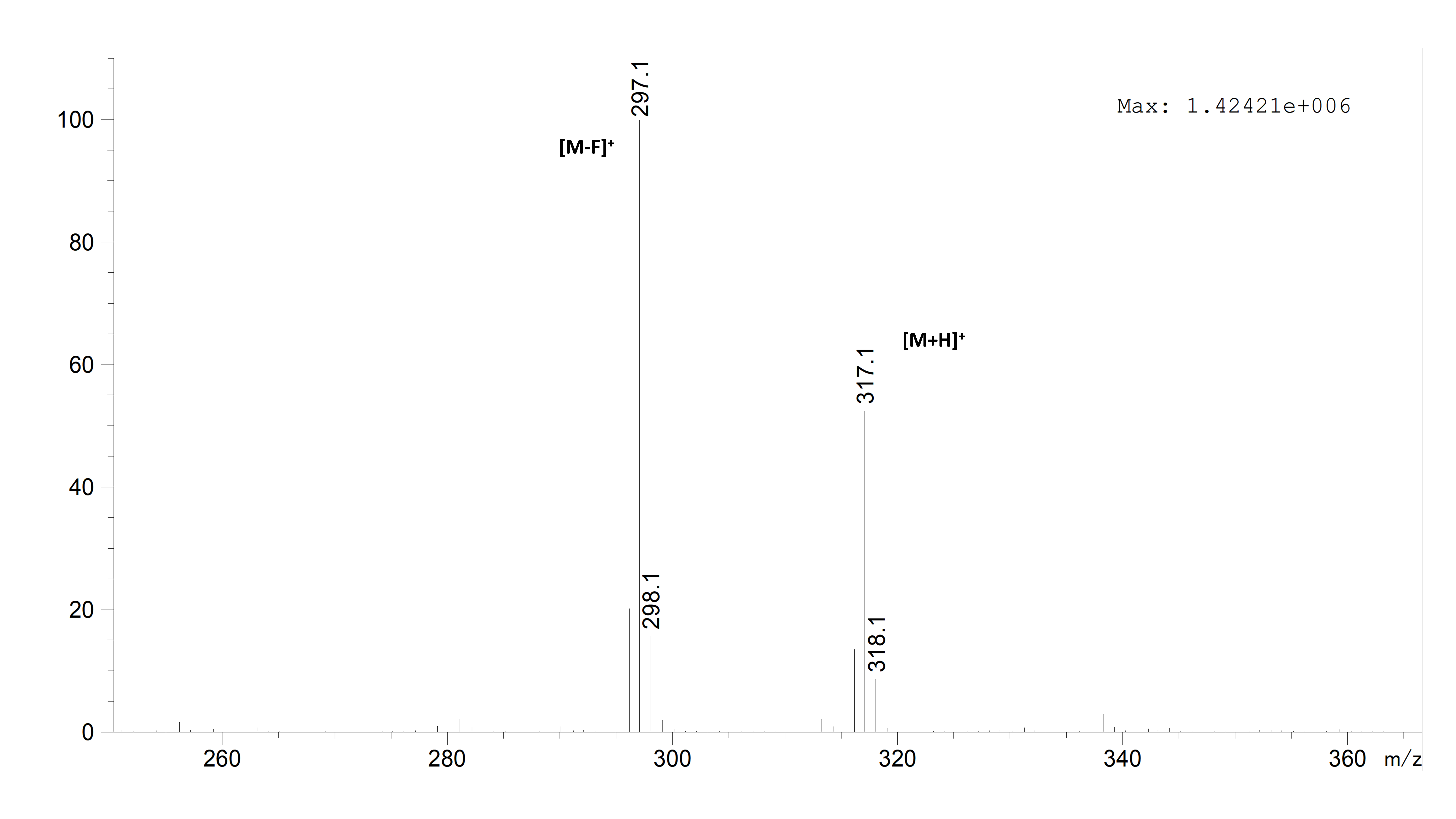


**Fig. S11.** MS spectrum of Compound 1.

**
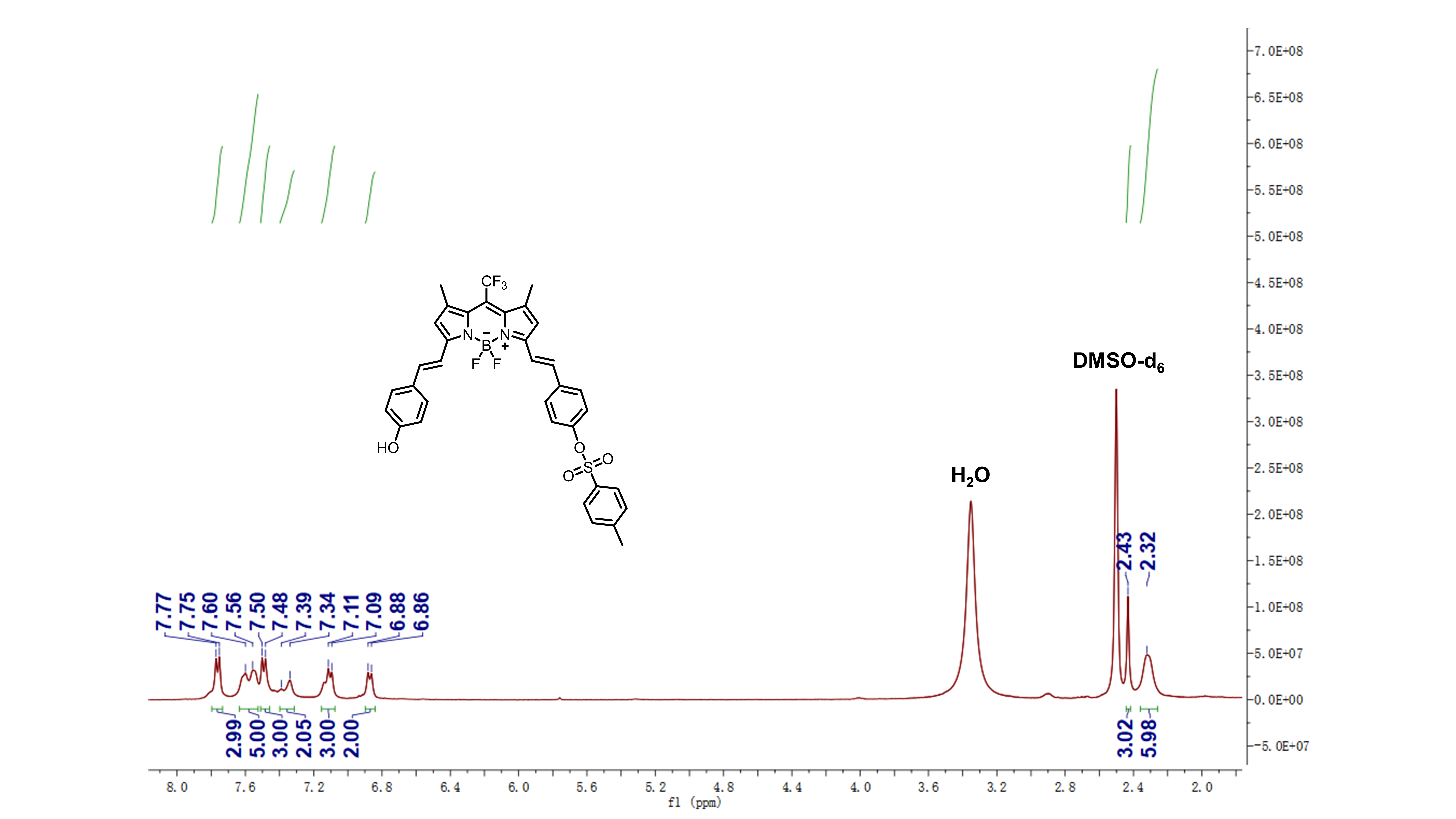
**

**Fig. S12** ^1^H NMR spectrum of Compound ER-BDP (400 MHz, DMSO-d_6_).

**
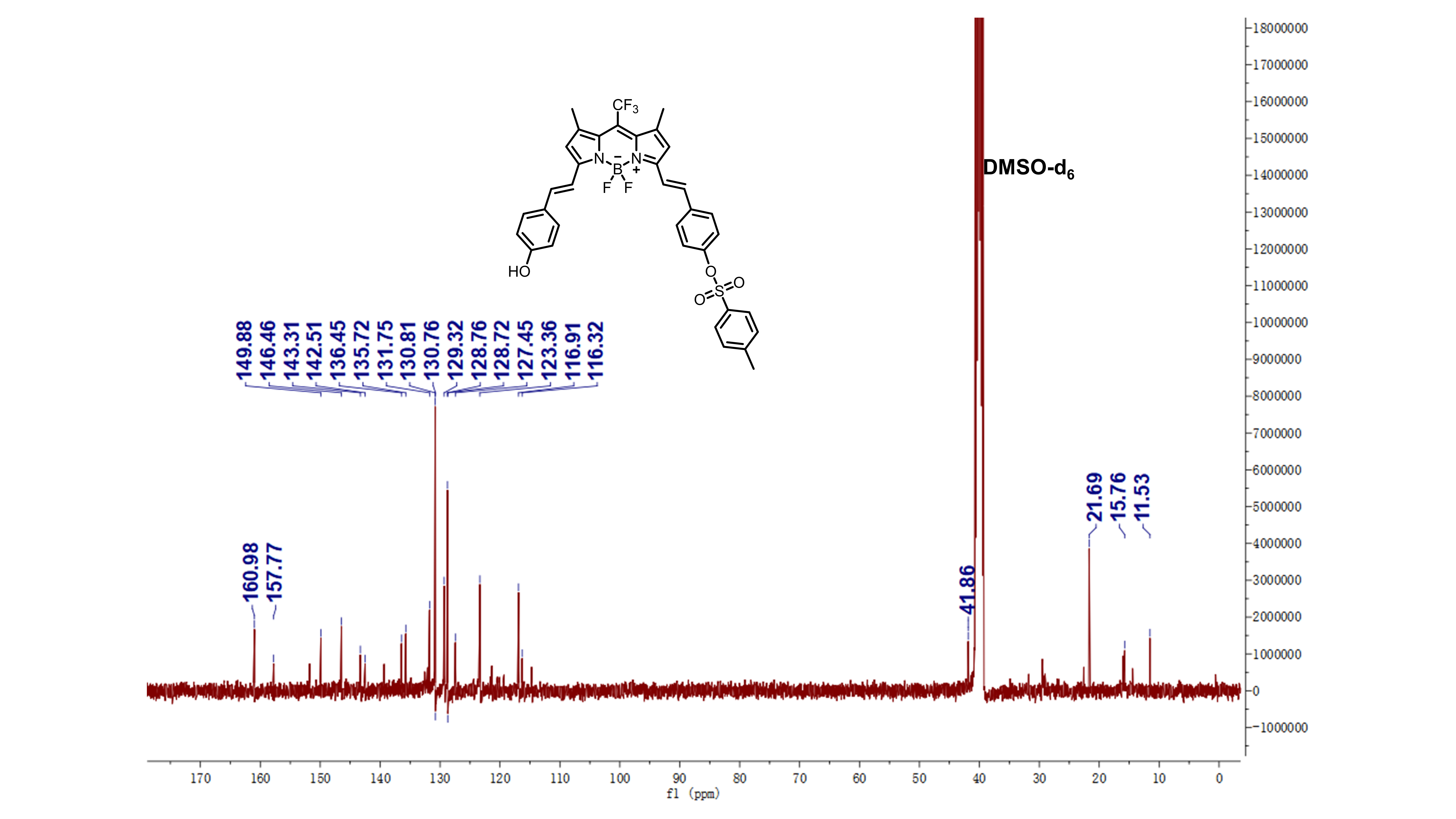
**

**Fig. S13.** ^13^C NMR spectrum of Compound ER-BDP (400 MHz, DMSO-d_6_).

**
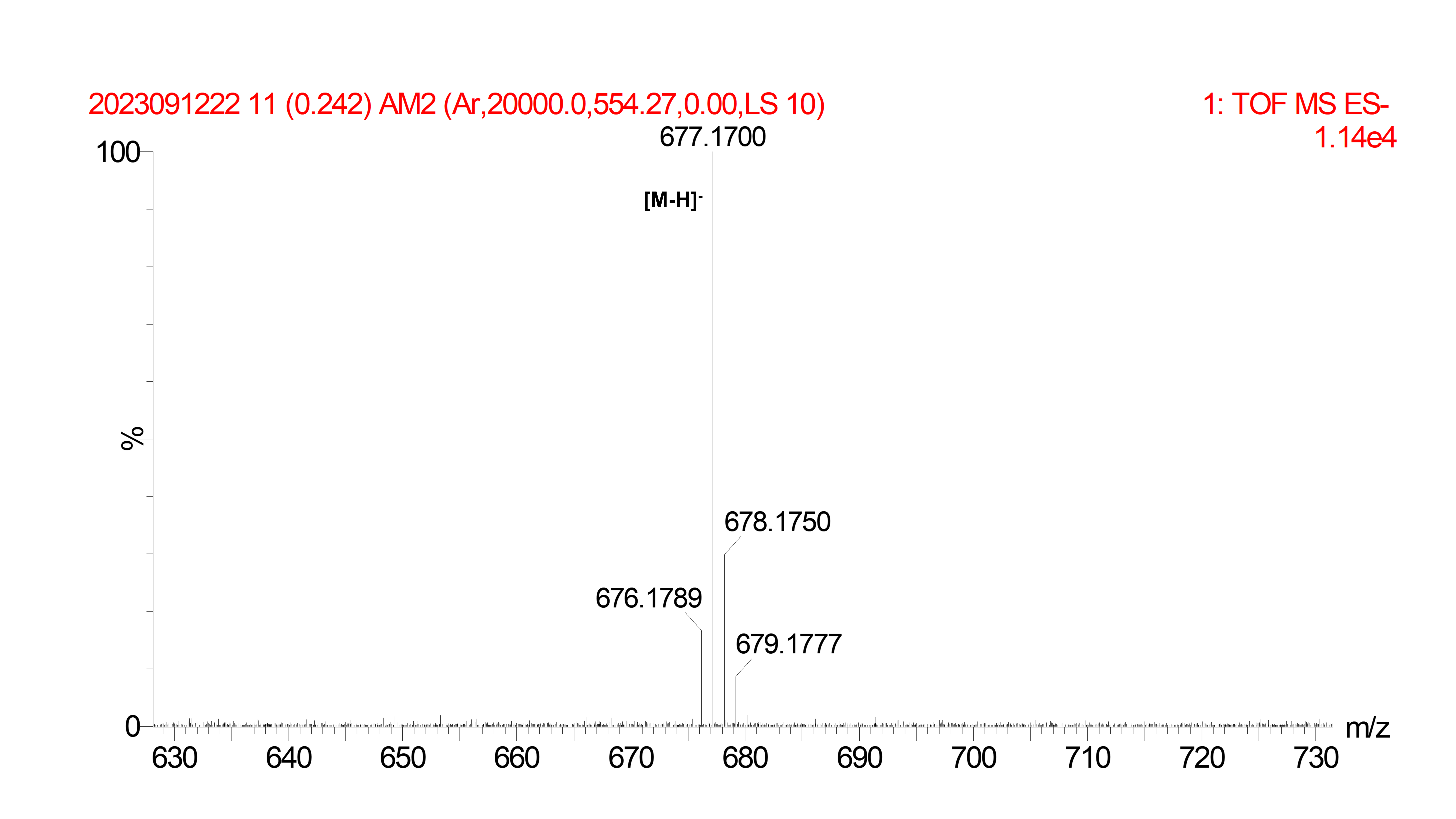
**

**Fig. S14.** HRMS spectrum of Compound ER-BDP.

**Reference**

1. D. M. Xi, M. Xiao, J. F. Cao, L. Zhao, N. Xu, S. R. Long, J. L. Fan, K. Shao, W. Sun, X. Yan and X. J. Peng, *Adv. Mater.* **2020**, 32, e1907855.
